# Seven new species of *Begonia* (Begoniaceae) in Northern Vietnam and Southern China

**DOI:** 10.3897/phytokeys.94.23248

**Published:** 2018-01-29

**Authors:** Wen-Hong Chen, Sirilak Radbouchoom, Hieu Quang Nguyen, Hiep Tien Nguyen, Khang Sinh Nguyen4, Yu-Min Shui

**Affiliations:** 1 Key Laboratory for Plant Diversity and Biogeography of East Asia, Kunming Institute of Botany, Chinese Academy of Sciences, Kunming 650201, China; 2 Southeast Asia Biodiversity Research Institute, Chinese Academy of Sciences, Yezin, Nay Pyi Taw 05282, Myanmar; 3 Center for Plant Conservation of Vietnam (CPC), Vietnam Union of Science and Technology Associations, 25/32 Lane 191, Lac Long Quan Rd., Hanoi, Vietnam; 4 Institute of Ecology and Biological Resources, Vietnam Academy of Science and Technology, 18 Hoang Quoc Viet Road, Hanoi, Vietnam; 5 University of Chinese Academy of Sciences, Beijing 100049, China

**Keywords:** *Begonia*, *Begonia* sect. *Coelocentrum*, *Begonia* sect. *Diploclinium*, *Begonia* sect. *Leprosae*, *Begonia* sect. *Sphenanthera*, China, new species, Vietnam

## Abstract

Since 2016, KIB (Kunming Institute of Botany) and CPC (Centre for Plant Conservation of Vietnam) have conducted several surveys in the transboundary karst regions in Northern Vietnam and Southern China and seven new species in the genus *Begonia* Linn. (Begoniaceae) are firstly described. Amongst them, two species, *Begonia albopunctata* Y.M. Shui, W.H. Chen & H.Q. Nguyen and *B. erectocarpa* H.Q. Nguyen, Y.M. Shui & W.H. Chen, respectively belong to section Sphenanthera with berry fruits and section Leprosae with clavate berry fruits; four species, *B. gulongshanensis* Y.M. Shui & W. H. Chen, *B. minissima* H.Q. Nguyen, Y.M. Shui & W.H. Chen, *B. mollissima* Y.M. Shui, H.Q. Nguyen & W.H. Chen, *B. rhytidophylla* Y.M. Shui & W.H. Chen, belong to section Coelocentrum with parietal placentation; one species, *Begonia bambusetorum* H.Q. Nguyen, Y.M. Shui & W.H. Chen, belongs to section Diploclinium with 3-loculed ovary and capsules. The diagnostic characters of these species are described and illustrated in the text and photographs.

## Introduction

**Figure 1.**
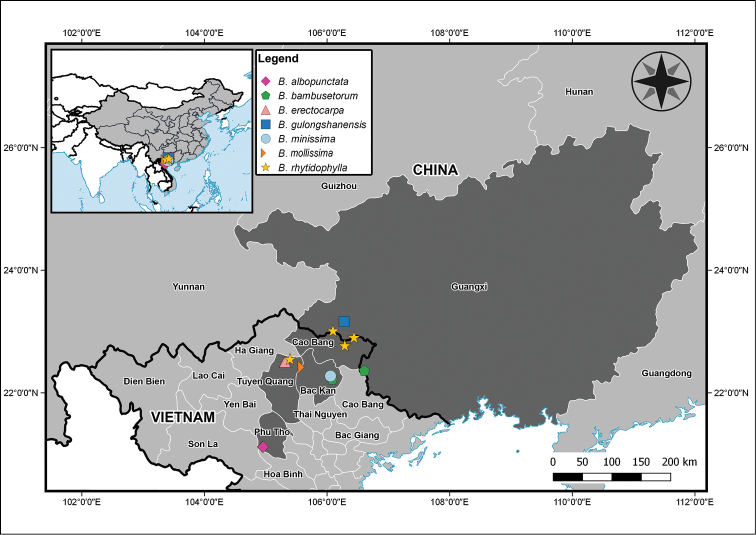
The geographical distribution of the seven new species of *Begonia* (Begoniaceae) from North Vietnam and South China under QGIS version 2.14.19.

The Tonkin region is one of the biodiversity hotspots worldwide (Takhtajan 1986; Myers et al. 2000) that include Northern Vietnam and Southern China (Fig. 1). This region is characterised by the massive area of the limestone landform and the lowland less than 1200 m elevation (Fang et al. 1994; Averyanov et al. 2003). The floristic region mainly covers the northernmost region from the North-west to North-east in Vietnam (Takhtajan 1986; Averyanov et al. 2003) that borders with the South-western Guangxi and South-eastern Yunnan in China (Wu and Wu 1998). In the last ten years, a large number of new species in different families have been discovered in the region, such as Begoniaceae, Gesneriaceae, Magnoliaceae etc. (Averyanov and Nguyen 2012; Chen et al. 2017; etc.).

Since 2016, for nearly three months, transboundary surveys have been conducted in Northwestern and Northeastern Vietnam (Bac Kan, Cao Bang, Lao Cai, Phu Tho, Tuyen Quang) and Southern China (Southwestern Guangxi and Southeastern Yunnan). After review of the type specimens and taxonomic publications within the regions (Gagnepain 1921; Irmscher 1939; Ho 1991; Nguyen 2004; Shui and Chen 2005; Truong et al. 2005; Nguyen and Tebbit 2006; Kiew 2007; Nguyen et al. 2010; Peng et al. 2014, 2015a,b; Hughes et al. 2015), seven new species of *Begonia* have been confirmed, and their sectional positions designated here (Doorenbos et al. 1998; Shui et al. 2002). Due to the taxonomic complexity of the genus (Thomas et al. 2011; Chung et al. 2014), the DNA samples of the above new species have also been collected and some possible different opinions will be issued about their systematic position in the broad context in the future.

## Taxonomy

### Begonia albopunctata

Taxon classificationPlantaeORDOFAMILIA

Y.M.Shui, W.H.Chen & H.Q.Nguyen sp. nov.

urn:lsid:ipni.org:names:77175484-1

Fig. 2

Begonia sect. Sphenanthera (Hassk) Warb.

#### Remarks.

The new species is similar to *B. hahiepiana* H.Q. Nguyen & Tebbitt in broadly ovate asymmetric leaves with slightly truncate or round apex and the corned berry fruit, but different from it in the broadly ovate or rotund leaves (vs. ovate), almost flat adaxial surface of leaves (vs. puckered), glabrous outer surface of petals (vs. with red trichomes), 4-loculed and glabrous berry fruit covered by dense white spots (vs. 3-loculed and pubescent fruit covered by the lax white spots).

#### Type.

VIETNAM. Phu Tho Province, Xuan Son county, Xuan Son National Park, 21°07’05”N, 104°57’09”E, 478 m a.s.l., 8 April 2016, *Y.M. Shui, W.H. Chen, C. Liu, H.Q. Nguyen, H.T. Nguyen, N.Q. Chuong CK 0916* (holotype, KUN!; isotype, CPC!=Herbarium of the Centre for Plant Conservation, Vietnam Union of Science and Technology Associations, Hanoi).

#### Herb, rhizomatous.

Rhizome: 4–6 cm long, 0.5–1 cm in diam. Stipule pale brown to reddish, triangular, glabrous, 0.7–1 cm × 0.2–0.4 cm, margin entire, apex acuminate. Leaves: petiole terete, 7.5–11 cm long, 1–2 mm in diam., densely covered by red lanes; blade greenish, asymmetric, broadly ovate or rotund, 8–14 × 6.5–9 cm; base cordate; apex subacute to obtuse; margin denticulate, short ciliate; adaxially greenish, almost flat, slightly glabrous and extremely sparely strigillose between secondary veins; abaxially red, veins densely red pubescent; venation palmate, 5–6 primary veins, secondary veins brunching dichotomous, tertiary veins reticulate. Inflorescence: dichasial cyme, peduncle erect, 6–9 cm long, red or brownish villous; bracts caducous, triangular to lanceolate, reddish, 5–7 × 2–3 mm, apex acuminate, margin entire. Staminate flower: pedicels 0.5–1.5 cm long; tepals 4, white, adaxially subglabrous, abaxially glabrous; outer tepals 2, broadly ovate, 0.7–1.5 × 0.6–1.2 cm; inner tepals 2, white, oblanceolate to oblong, 0.8–1.6 × 0.2–0.5 cm, base cuneate to rounded, apex acute to obtuse, margin entire; androecium actinomorphic, stamens numerous; filaments fused at base; anthers yellow, obovate, 1–1.2 mm long, apex convex, shorter than filaments, with longitudinal slits. Pistillate flower: pedicel 0.4–0.7 cm long; tepals 5, white; outer tepals 3, broadly ovate, 0.8–1.2 × 0.4–0.6 cm, apex obtuse to rounded, margin entire; inner tepals 2, ovate, 0.5–0.7 × 0.4–0.6 cm, apex obtuse to rounded, margin entire; styles 3, free; stigmas bifid with twisted bands; ovary reddish, 0.2–0.3 cm long, 0.2–0.4 cm in diam., with white papillose and 4 thickened corns; placentation axile, 4-loculed, placentae 2 each locule. Fruit berrylike, white papillose, wingless and with 4 thickened rib-like horns.

#### Phenology.

Flowering in April–May, fruiting in April–June.

#### Etymology.

The epithet refers to the white spots on the fruit surface.

#### Habitat.

The species only grows in deep ground amongst rocks in limestone forests.

#### Distribution.

The species occurs exclusively in Phu Tho Province, Xuan Son country, Xuan Son National Park of Vietnam.

#### Additional examined specimens.

VIETNAM. Phu Tho Province, Xuan Son county, Xuan Son National Park, 21°07’01”N, 104°57’29”E, 438 m a.s.l., 8 April, 2016, *Y.M. Shui, W.H. Chen, C. Liu, H.Q. Nguyen, H. T. Nguyen, N. Q. Chuong CK 0918* (KUN, CPC).

#### Note.

Currently six species from section Sphenanthera are recognised as occurring in Vietnam: *Begonia acetosella* Craib, *B. balansana* Gagnep., *B. handelii* Irmscher, *B. longifolia* Blume, *B. ceratocarpa* S.H. Huang & Y.M. Shui and *B. hahiepiana* H.Q. Nguyen & Tebbitt. Although this new species grows together with *B. hahiepiana*, it is easily distinguished by the glabrous and 4-loculed berry fruit, which has been discussed in the above diagnostic description. Within the Vietnamese species of the section, the stemless habit, the narrow inflorescence and the small horned appendage of the berry fruit indicate that the new species is also similar to *Begonia ceratocarpa* and *B. balansana*. The detailed comparison reveals that it is more similar to *B. ceratocarpa* with 3–4-loculed fruits than *B. balansana* with 6–7-loculed fruit. It is different from *B. ceratocarpa* in the broadly ovate or rotund leaves (*vs.* ovate), the unlobed leaf blade (*vs.* slightly lobed), round leaf top (*vs.* acuminate leaf top), petioles with red pubes (*vs.* with brown 1anes), 5 female tepals (*vs.* 3), densely white-spotted surface of berry fruit (*vs.* sparely spotted), truncate fruit top without a beak (*vs.* an acute fruit top with a beak).

**Figure 2.**
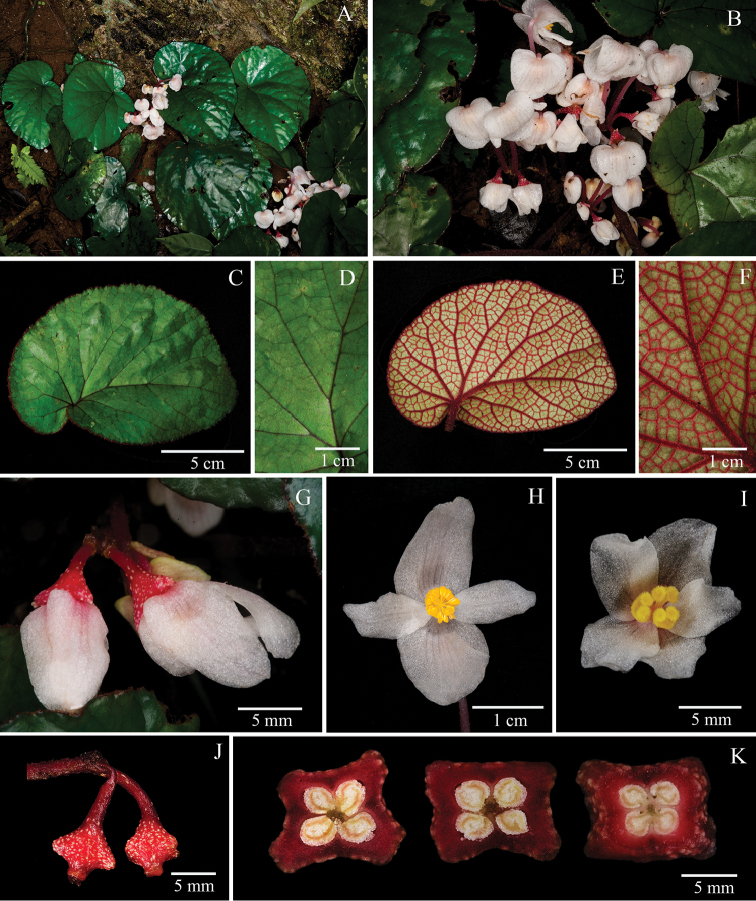
*Begonia albopunctata* Y.M. Shui, W.H. Chen & H.Q. Nguyen **A** Habitat **B** Inflorescence **C** View of adaxial leaf **D** Close-up of adaxial leaf **E** View of abaxial leaf **F** Close-up of abaxial leaf **G** Pistillate flower, side view **H** Staminate flower, face view **I** Pistillate flower, face view **J** Fruits **K** Serial cross sections of ovary. (**A–J** photographs by Y.M. Shui; **K** by S. Radbouchoom).

### Begonia bambusetorum

Taxon classificationPlantaeORDOFAMILIA

H.Q.Nguyen, Y.M.Shui & W.H.Chen sp. nov.

urn:lsid:ipni.org:names:77175485-1

Fig. 3

Begonia sect. Diploclinium A. DC.

#### Remarks.

The new species is similar to *Begonia sinovietnamica* C. Y. Wu in their habit and distribution (Fig. 1; Fig. 2), but different from the latter species in expanding villous hairs on petiole and peduncles (vs. pubescent), 2 petals of the male flowers (vs. 4 petals), 2 petals of the female flowers (vs. 5 rarely 4 petals).

#### Type.

VIETNAM. Bac Kan Province, Nari county, An Tinh committee, 22°12’39”N, 106°05’02”E, 285 m a.s.l., in the forests of Bamboo around streams, flowers pinkish, 23 April 2016, *Y.M. Shui, W.H. Chen, C. Liu, H.Q. Nguyen, H.T. Nguyen, N.Q. Chuong CK 1296* (holotype, KUN!; isotype, CPC!)

#### Herb, rhizomatous.

Rhizome: densely villous, 9–19 cm long, 0.5–1 cm in diam. Stipule reddish, triangular, 0.8–1.5 × 0.4–0.7 cm, densely villous outside, glabrous inside, margin entire and ciliate. Leaves: petiole terete, greenish to reddish, villous, 14–19 cm long, 1.5–3 mm in diam.; blade greenish, asymmetric, ovate to widely ovate, 11–17 × 8.5–11.5 cm, slightly rugose; base cordate, apex caudate, margin serrulate and long ciliate; adaxially greenish, sparsely brevi-setose, abaxially greenish, sparely villous and densely strigose on veins; venation palmate, 6–8 primary veins, secondary veins brunching dichotomous, tertiary veins slightly reticulate, veins prominent on both sides. Inflorescence: dichasial cyme, peduncle erect, 4–10 cm long, villous; bracts caducous, triangulate to lanceolate, reddish, 5–7 × 2–3 mm, adaxially red villous, apex acuminate, margin serrate and ciliate, abaxially glabrous. Staminate flower: pedice 1–1.5 cm long; tepals 2, white to pink, adaxially red villous, abaxially glabrous; tepals 2, widely ovate, 1.5–2 × 0.7–1 cm, apex obtuse to rounded, margin entire; androecium actinomorphic, stamens numerous, filaments almost free, 1–2 mm long, anthers yellow, obovate, 1–1.2 mm long, apex convex, shorter than filaments, with two lateral longitudinal slits. Pistillate flower: pedicel 1–1.5 cm long; tepals 2, white to pink, adaxially red villous, abaxially glabrous; outer tepals 2, widely ovate, 1–1.5 × 0.7–1.2 cm, apex obtuse to rounded, margin entire; styles 3, free, stigmas bifid with twisted bands; ovary white, sparely red villous; placentation axile with 2-segments per locule. Capsule nodding, 0.35–0.5 cm long, 0.27–0.31 cm in diam. (wings excluded), with 3 subequal wings; abaxial wing 0.6–1 × 0.3–0.7 cm; lateral wings 0.4–0.7 × 0.5–0.7 cm.

#### Phenology.

Flowering in May–June, fruiting in June–August.

#### Etymology.

The epithet refers to the habitat of the new species: the bamboo forests along streams.

#### Habitat.

The new species just grows in bamboo forests along watersides.

**Distribution.** The species occurs both in Nari county of Bac Kan Province in Vietnam and in Longzhou county of Guangxi, China.

#### Additional specimens examined.

CHINA. Guangxi Zhuang Autonomous Region, Longzhou Xian, Chunxiu community, 22°21’22”N, 106°36’34”E, alt. 460 m a.s.l., November, 2016, cultivated in Horticulture Nursery of Beijing Florascape Company, in flowers, 1 October, 2017, *Y. M. Shui and S. W. Guo* BE-004 (KUN).

#### Note.

The new species seems to be a member of the first group with rhizome and without erect stem in Begonia sect. Diploclinium A. DC. (Doorenbos et al. 1998). Within the group, it is more similar to *Begonia sinovietnamica* in the locality and habit than the other, but different in the hairs of plants and morphology of the flowers. Additionally, its reticulate nerves on the upper surface of leaves are more obvious than those in *B. sinovietnamica*, which can easily be examined in living plants instead of in dry specimens.

**Figure 3.**
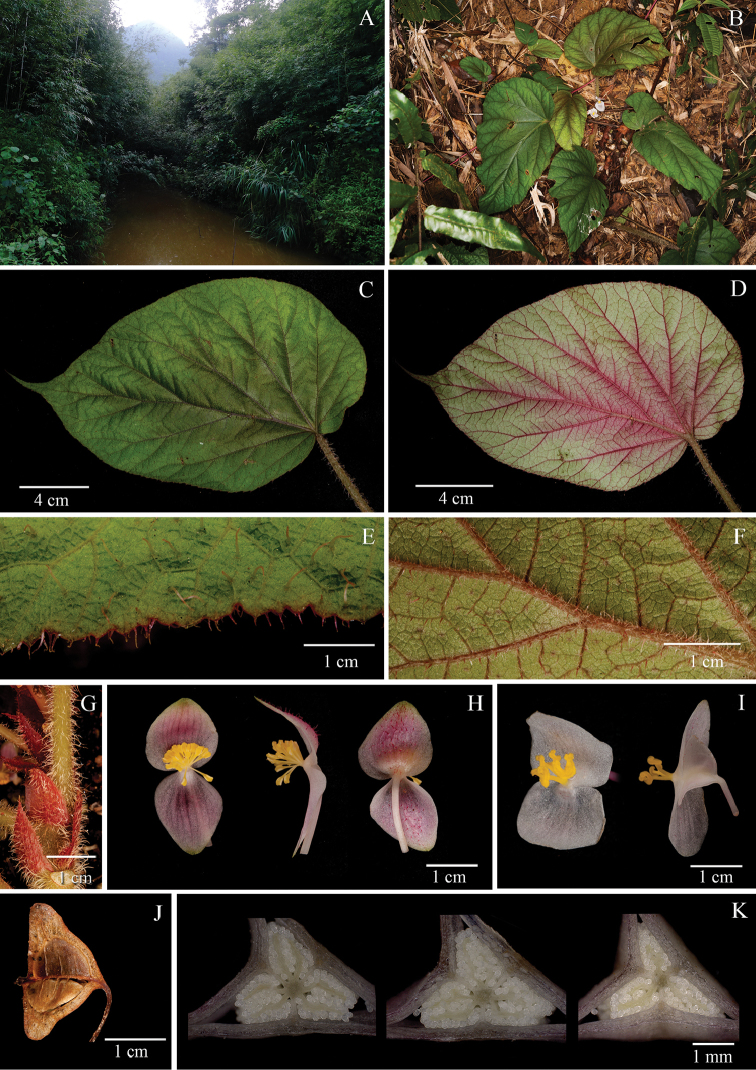
*Begonia bambusetorum* H.Q. Nguyen, Y.M. Shui & W.H. Chen **A** Habitat **B** Plant **C** View of adaxial leaf **D** View of abaxial leaf **E** Close-up of adaxial leaf **F** Close up of abaxial leaf **G** Stipule and petiole **H** Staminate flower, face view, side view and dorsal view **I** Pistillate flower, face view and side view **J** Fruits **K** Serial cross sections of ovary. (**A–D** photographs by Y.M. Shui; **E–K** by S. Radbouchoom).

### Begonia erectocarpa

Taxon classificationPlantaeORDOFAMILIA

H.Q.Nguyen, Y.M.Shui & W.H.Chen sp. nov.

urn:lsid:ipni.org:names:77175486-1

Fig. 4

Begonia sect. Leprosae Y. M. Shui

#### Remarks.

The new species is similar to *Begonia longicarpa* K.Y. Guan et D. K. Tian in the clavate berry fruit, but differs from it in its hispid petiole (vs. pubescent), five female petals (vs. three) and short segments of placentation per locule (vs. long segments).

#### Type.

VIETNAM. Tuyen Quang province, Lam Binh district, Thuong Lam community, in the secondary tropical evergreen lowland rainforest and broad-leaved forests on the slopes and ridge of crystalline limestone hills with highly eroded earth, grow on ground soil, male and female flowers white, fruit berry, green or pink, 22°30'05"N, 105°18'11"E, 484 m a.s.l., 11 October 2017, *H.Q. Nguyen,H.T. Nguyen, K. S. Nguyen, N.Q. Chuong CPC 8463* (holotype, KUN!; isotype, CPC!).

#### Herb, rhizomatous.

Rhizome: 3–5 cm long, 1.0–1.7 cm in diam. Stipule triangular, 0.8–1×0.2–0.4 cm, pale brown to reddish, margin entire, apex acuminate, adaxially glabrous, abaxially red hirsute. Leaves all basal, alternate; petiole terete, 9–15 cm long, 2–5 mm in diam., densely reddish hirsute; blade dark green or brown, asymmetric, broadly ovate, 7–14× 3.5–9.0 cm, base cordate, oblique, apex subacute to obtuse, margin denticulate, short ciliate, adaxially dark green, almost glabrous, usually with white spots, abaxially dark red, densely red lanate on veins; venation palmate, 6–7 primary veins, secondary veins brunching dichotomous, tertiary veins obviously reticulate, densely velutinous on veins. Inflorescence dischasial cyme, axillary, peduncle 3–5 cm, erect, red to brownish villous; bracts pale greenish, triangular to lanceolate, 1–1.5× 2–3 mm, margin dentate and ciliate, apex acuminate. Staminate flower: pedicel 2.2–3.5 cm, glabrous above the middle, villous below the middle; tepals 4, white-pinkish, glabrous; outer tepals 2, ovate, 1.2–1.5× 0.6–1 cm, apex acute, base cuneate, margin entire; inner tepals 2, oblanceolate to oblong, white-pinkish, 0.8–1.6× 0.2–0.4 cm, base cuneate, apex acute, margin entire; stamens numerous, filaments free, anthers yellow, obovate, 1–1.2 mm long, apex rounded, shorter than filament, with longitudinal slits. Pistillate flower: pedicel 8–1.6 cm long, hirsute; tepals 5, white to pinkish, glabrous; outer tepals 3, ovate, 0.8–1.0× 0.3–0.5 cm, apex obtuse to rounded, margin entire; inner tepals 2, ovate, 0.6–1.0× 0.2–0.4 cm, apex obtuse to rounded, margin entire; styles 3, free, stigmas bilobed, with twisted band; ovary green or pinkish, 1.5–2.0 cm long, 0.3–0.5 cm in diam., cylindric, wingless, puberulent; placentation axile, 3-locular, placentae partly branching 2–4 each locule. Fruit berrylike, wingless.

#### Phenology.

Flowering in October–December, fruiting in November–January next year.

#### Etymology.

**Figure 4.**
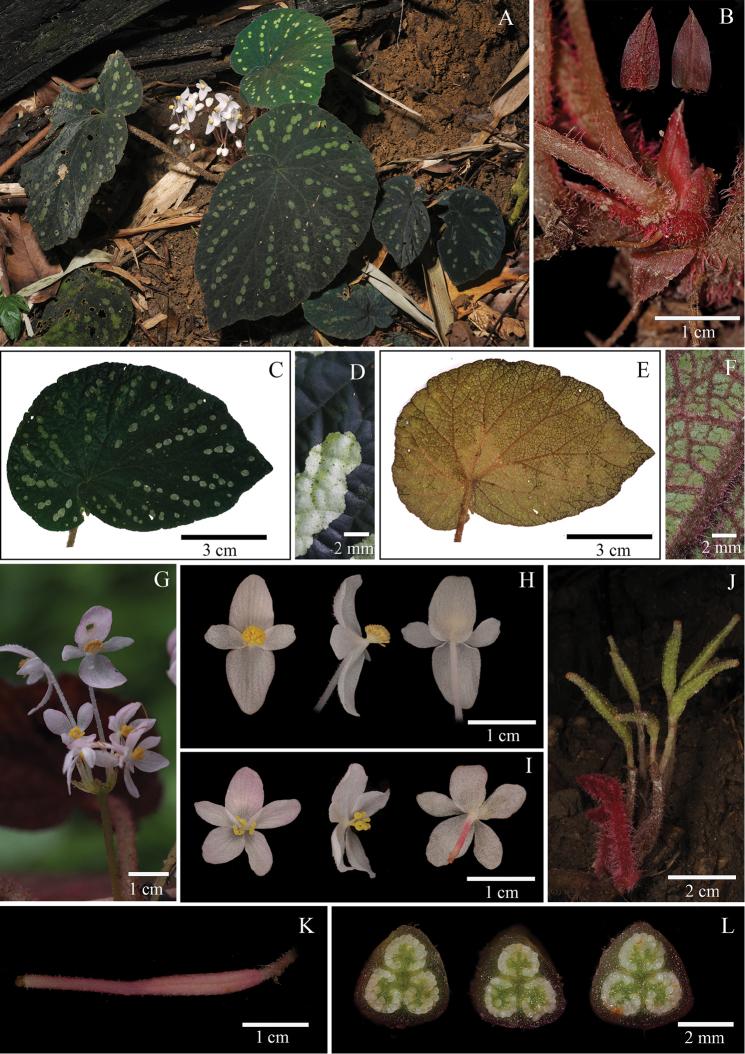
*Begonia erectocarpa* H.Q. Nguyen, Y.M. Shui & W.H. Chen **A** Habitat **B** Stipule, dorsal view and face view **C** View of adaxial leaf **D** Close-up of adaxial leaf **E** View of abaxial leaf **F** Close-up of abaxial leaf **G** Inflorescence **H** Staminate flower, face view, side view and dorsal view **I** Pistillate flower, face view, side view and dorsal view **J** Fruits **K** Young fruit **L** Serial cross sections of ovary. (**A, C, E, H, I, J, K** photographs by S.W. Guo; **B, D, F, G** and **L** by H.Q. Nguyen).

The epithet refers to the upward fruit when nearly mature (Fig. 4-J). The erect case of fruit when nearly mature is unusual in the genus *Begonia*. The exceptional species is in some species in Begonia sect. Trachelocarpus (C. Müller) A. DC., such as *Begonia lanceolata* Vellozo in Brazil (Doorenbos et al. 1998; Tebbitt 2005).

#### Habitat.

The species just grows on soil within the secondary tropical evergreen lowland rainforest and broad-leaved forests on the slopes and ridge of crystalline limestone with highly eroded earth at an elevation 400–700 m above sea level.

#### Distribution.

The species occurs exclusively in Tuyen Quang Province in Vietnam.

#### Additional examined specimens.

VIETNAM. Tuyen Quang province, Lam Binh district, Thuong Lam community, 22°30'17"N, 105°18'48"E, 420 m a.s.l., 13 October 2017, in flower, *H.Q. Nguyen et al. CPC 8449* (KUN!; CPC!). The same locality, 22°30'06"N, 105°19'29"E, 255 m a.s.l., 29 November 2017, flower pinkish, *Y.M. Shui, W.H. Chen, S.W. Guo, H.Q. Nguyen,H.T. Nguyen, K. S. Nguyen, N.Q. Chuong CK1505* (KUN!; CPC!). The same place, 420 m a.s.l., 30 November 2017, in fruit, *Y.M. Shui, W.H. Chen, S.W. Guo, Q.H. Nguyen, T.H. Nguyen, S.K. Nguyen CK1513* (KUN!; CPC!).

#### Note.

The new species should be a member of Begonia sect. Leprosae Y.M. Shui according to its clavate berry fruit (Shui et al. 2002). In the section, it is more similar to *B. longicarpa* and *B. leprosa* Hance than the other species. It is similar to *B. longicarpa* in the habitat of ground soil and different mainly in the petal number (5 *vs.* 3), the hairs of petiole (hispid *vs.* pubescent) and the morphology of the segments of placentation per locule (irregular placenta segments vs. 2 regular placenta segments per locule). It is also similar to *B. leprosa* in the morphology of the fruit and hairs on the petiole, but differs mainly in the habitat (ground soil *vs.* limestone surface), the abaxial surface of leaves (obviously reticulate nerves *vs.* obscurely reticulate nerves) and the petal number of the female (5 *vs.* 4) and the morphology of placentation at the upper part of ovary (axile *vs.* parieta). As to the white spots on the adaxial leaf surface, it is somewhat similar to *Begonia gulinqingensis* S.H.Huang & Y.M.Shui in Begonia sect. Diploclinium, but different in the fruit (berry *vs.* capsule) (Fig. 4-J; Shui and Chen 2017).

### Begonia gulongshanensis

Taxon classificationPlantaeORDOFAMILIA

Y.M.Shui & W.H.Chen sp. nov.

urn:lsid:ipni.org:names:77175487-1

Fig. 5

Begonia sect. Coelocentrum Irmsch.

#### Remarks.

The new species is similar to *Begonia daxinensis* T. C. Ku, but different from it in its ovate leaves (vs. broadly ovate in *B. daxinensis*), slightly oblique leaf base (vs. extremely oblique), glandulose-villous on peduncles, pedicels, adaxial surface of exterior tepals and fruits (vs. glabrous, sparely eglandulose-pubescent), filaments connected to a half (vs. only at base), the smaller petals of male flowers (0.7–1.2 × 0.7–0.9 cm vs. 1.2–1.9 × 1–1.6 cm), the smaller petals of the female flowers (0.9 × 1.0 vs. 1–1.5 × 1–1.2 cm) .

#### Type.

CHINA. Guangxi Zhuang Autonomous Region, Jingxi county, Gulongshan, 23°19’44”N, 106°16’46”E, 286 m a.s.l., in the deep valley, growing on the moist surface of steep cliffs, flower pinkish, 18 February 2016, *Y.M. Shui et al. B2016-048* (holotype, KUN!).

#### Herb, rhizomatous.

Rhizome: 0.3–0.5 cm in diam. Stipule triangular, 0.2–0.4 × 0.6–0.8 cm, apex acute, adaxially subglabrous, abaxially hispid. Leaves all basal, alternate; petiole 5–9 cm long, glandular-hispid; blade greenish, asymmetric, ovate or ovate-lanceolate, 12–18 × 5–9 cm, base cordate, auricular, oblique or slightly oblique, apex caudate, margin serrulate; adaxially greenish, tubercular-setose, with white semi-circle in the middle and with dark red patches along the main nerves; abaxially greenish, glandular-pubescent along veinlets, sparsely villous and densely strigose on main veins; venation palmate, ca. 7 primary veins, secondary veins branching dichotomous, tertiary veins slightly reticulate, veins prominent adaxially. Inflorescence dichasial cyme, axillary, peduncle 5–9 cm, glandular-villous; bracts caducous, oblong-lanceolate, ca. 5 × 1 mm, apex acuminate, adaxially glabrous, abaxially long setulose. Staminate flower: pedicel 1.1–1.8 cm long, glandular-villous; tepals 4, pinkish to pink; outer tepals 2, widely ovate, 0.7–1.2 × 0.7–0.9 cm, apex rotund, margin entire, adaxially glabrous, abaxially glandular-villous; inner tepals 2, narrowly oblong or obovate, 0.6–1.0 × 0.2–0.25 cm, apex acute; androecium actinomorphic; stamens numerous, filaments connected to a half, ca. 1 mm long; anthers yellow, obovate, ca. 0.5 mm long, apex emarginate. Pistillate flower: pedicel ca. 1.1 cm long, glandular-villous; tepals 3, pinkish or pink; outer tepals 2, widely ovate, ca. 0.9 × 1.0 cm, apex rotund, margin entire, adaxially glabrous, abaxially glandular-villous; inner tepal 1, oblong, 5–6 × 2–3 mm, base cuneate, apex acute, margin entire; styles 3, fused at base; stigmas spiralled, papillose; ovary green, elliptic, 0.8–0.9 cm long, glandular-villous; placentation parietal, 2-segmented per carpel. Capsule nodding, 3-winged unequally; major wing ca. 3.5 mm long, lateral wings ca. 2.1 mm long.

#### Phenology.

Flowering in February–May, fruiting in May–June.

#### Etymology.

The epithet refers to the locality of the type specimens.

#### Habitat.

The species only grows on the moist surface of shady cliffs at the entrance to shallow caves in a deep valley.

#### Distribution.

The species occurs exclusively in Jingxi county of Guangxi in China.

#### Note.

In Begonia sect. Coelocentrum, the new species is similar to the population of *B. daxinensis* with a white area on the leaves, but differs mainly in its long glandular hairs (Wu and Ku 1997; Ku et al. 2004). In the latter species, the petals are covered abaxially by brown pubescence and are much larger than those of the new species. As to the lanceolate and colourful leaves, the new species is similar to *Begonia locii* C.-I Peng, C. W. Lin & H. Q. Nguyen, but different in the absence of glandular hairs of flowers (Peng et al. 2015a). Furthermore, as to the glandular pubes, the new species is also similar to *B. filiformis* Irmsch., but differs in the subglabrous adaxial surface and laxly pubescent abaxial surface of leaves (*vs.* densely pubescent adaxial and abaxial surface of leaves in the latter).

**Figure 5.**
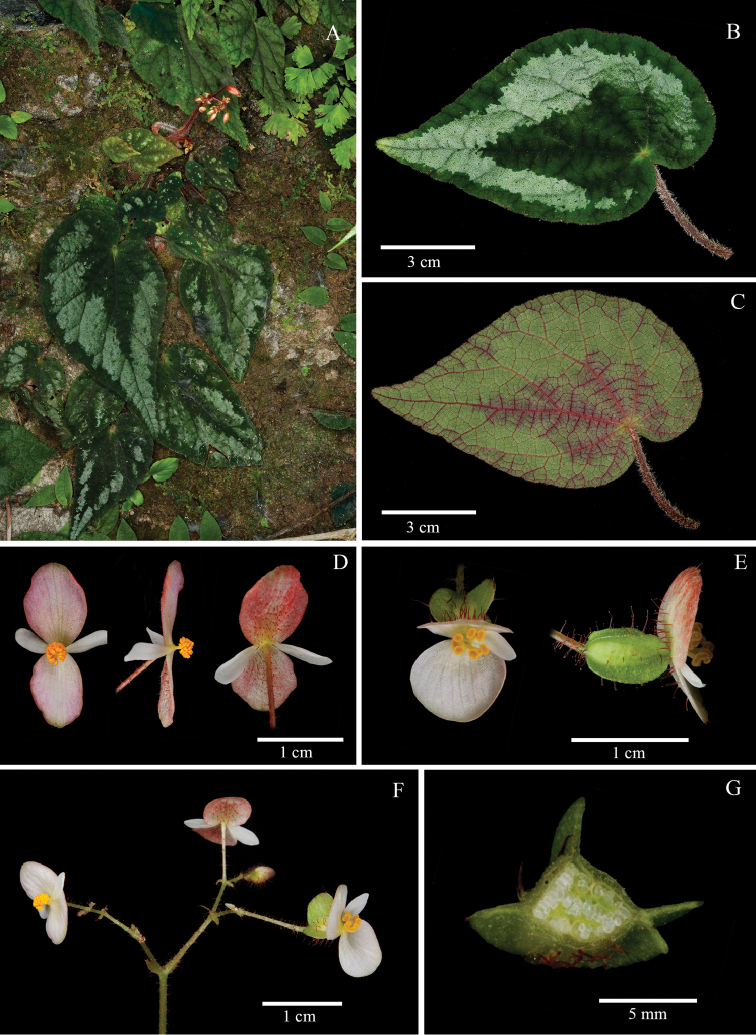
*Begonia gulongshanensis* Y.M. Shui & W.H. Chen **A** Habitat **B** View of adaxial leaf **C** View of abaxial leaf **D** Staminate flower, face view, side view and dorsal view **E** Pistillate flower, face view and side view **F** Inflorescence **K** Cross section of ovary in the middle part. (All photographs by Y.M. Shui).

### Begonia minissima

Taxon classificationPlantaeORDOFAMILIA

H.Q.Nguyen, Y.M.Shui & W.H.Chen sp. nov.

urn:lsid:ipni.org:names:77175488-1

Fig. 6

Begonia sect. Coelocentrum Irmsch.

#### Remarks.

The new species is similar to *Begonia rugosula* Aver. in the small leaves and climbing habit, but different from it in the round leaf top (vs. acute leaf top), flat surface on the adaxial surface of the leaves (vs. uneven surface in *B. rugosula*), ovate interior petals (vs. narrowly obovate or narrowly obovate), 2-branching stigma (vs. slightly spherical).

#### Type.

VIETNAM. Bac Kan Province, Nari county, Kim Hy National reserve, 22°16'22"N, 106°03'23"E, 504 m a.s.l., 18 April 2016, in flowers, *Y.M. Shui, W.H. Chen, C. Liu, H.Q. Nguyen, H.T. Nguyen, N.Q. Chuong CK1210* (holotype, KUN!; isotype, CPC!).

#### Small herb, rhizomatous.

Rhizome: slender, 2.5–7 mm long, 1.5–2 mm in diam. Stipule reddish, triangular, subglabrous, 1–1.8 × 8–1.2 mm wide. Leaves: petiole reddish, erect, villous, 1.5–3.5 cm long, 0.5–1 mm in diam.; blade asymmetric, widely ovate or rotund, 2.2–2.6 × 1.6–1.8 cm, base cordate, apex obtuse to acute, margin slightly serrulate, ciliate; adaxially dark green with white line or stripes along veins, strigose; abaxially slightly greenish striated and short strigose along primary and secondary veins; venation palmate, 4–5 primary veins, 2 or 3 secondary veins brunching dichotomous, tertiary veins reticulate. Inflorescence: dichasial cyme; peduncle 2–3.5 cm long, glabrous; bracts caducous, greenish, triangular, glabrous, apex acute, margin entire. Staminate flower: pedicel ca. 1 cm long; tepals 4, widely ovate, white inside, pinkish outside, both sides glabrous; outer tepals 2, broadly ovate, glabrous, base cuneate, apex obtuse or rounded, margin entire; inner tepals 2, ovate, base cuneate, apex acute to obtuse, margin entire; androecium actinomorphic, stamens numerous, filaments longer than anthers, fused at base; anthers yellow, obovoid, apex emarginate, with longitudinal slits. Pistillate flower: pedicel ca. 1 cm long; tepals 3, white, both sides glabrous; outer tepals 2, broadly ovate, ca. 5 × 6 mm, abaxially slightly pinkish with red spots, base rounded, apex obtuse or rounded, margin entire; inner tepal 1, elliptic to oblong, ca. 6 × 4 mm, base cuneate to rounded, apex obtuse, margin entire; styles 3, free, stigmas bifid with twisted bands; ovary slightly pinkish with red minute spots, ca. 5 mm long, ca. 3 mm in diam. (wings excluded), glabrous, 1-loculed; placentation parietal, 2-branched each placenta; Capsule nodding, trigonous-ellipsoid, subequally 3-winged; abaxial wing lunate, ca. 5 ×3 mm, slightly larger than lateral wings; lateral wings narrowly lunate, ca. 5 × 2 mm. Seeds ca. 0.15 mm long, oblong.

#### Phenology.

Flowering in April–May, fruiting in May–June.

#### Etymology.

The epithet refers to the small size of leaves.

#### Habitat.

The species just grows in limestone crevices of cliffs.

#### Distribution.

The species occurs exclusively in Nari county of Bac Kan Province in Vietnam.

#### Note.

**Figure 6.**
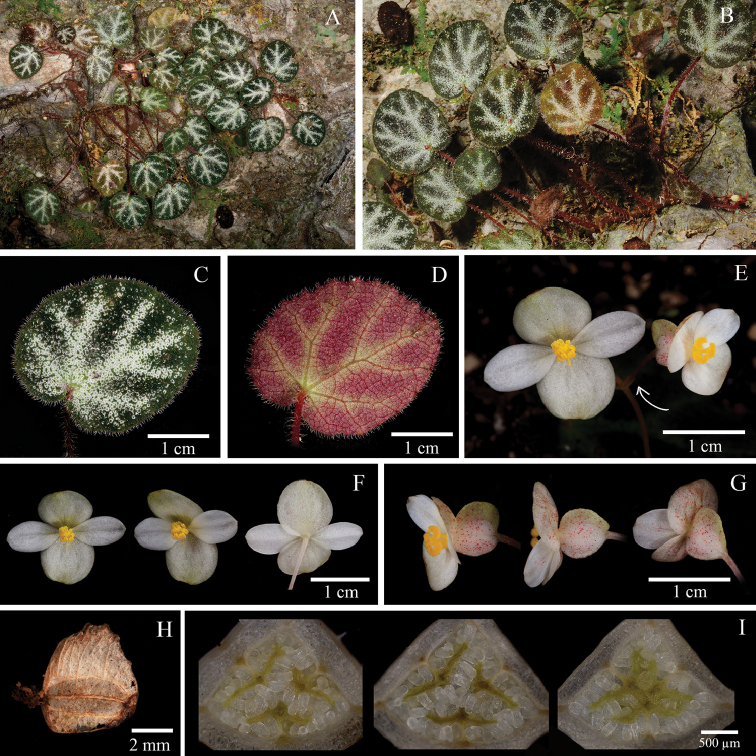
*Begonia minissima* H.Q. Nguyen, Y.M. Shui & W.H. Chen **A** Habitat **B** Plant with rhizome **C** View of adaxial leaf **D** View of abaxial leaf **E** Inflorescence **F** Staminate flower, face view, side view and dorsal view **G** Pistillate flower, side views and dorsal view **H** Capsule **I** Serial cross sections of ovary. (**A–D** photographs by Y.M. Shui; **E–I** by S. Radbouchoom).

In Begonia sect. Coelocentrum, the new species is one of the smallest species in morphology. Its closest species with limited morphology is *Begonia regosula* Aver. also from Bac Kan Province (Averyanov and Nguyen 2012). In the new species, however, the wider interior petals and the obvious bifid stigma can separate it from *B. regosula* (Fig. 6).

### Begonia mollissima

Taxon classificationPlantaeORDOFAMILIA

Y.M.Shui, H.Q.Nguyen & W.H.Chen sp. nov.

urn:lsid:ipni.org:names:77175489-1

Fig. 7

Begonia sect. Coelocentrum Irmsch.

#### Remarks.

The new species is similar to *Begonia guangxiensis* T.C. Ku in the dense white villous hairs on the whole plants, differs from the latter in the shorter hairs of plants (2.1–2.4 mm vs. 3.4–4.0 mm), its one obtuse leaf lobe (vs. 3–4 acute leaf lobes in B. guangxiensis), subequal three wings of the fruit (vs. unequal three wings), narrowly semi-lunar major wings (vs. broadly square major wings).

#### Type.

VIETNAM. Bac Kan Province, Cho Ra county, Ba Be National Park, 22°24'56"N, 105°37'48"E, 235 m a.s.l., 18 April 2016, *Y.M. Shui, W.H. Chen, C. Liu, H.Q. Nguyen,H.T. Nguyen, N.Q. Chuong CK1183* (holotype, KUN!; isotype, CPC!).

#### Herb, rhizomatous.

Rhizome, densely white villous, 2–3 cm long, 0.7–1 cm in diam. Stipule green, 1.5–2 × 1–1.5 cm, widely ovate, apex acute to obtuse, margin ciliate. Leaves: petiole erect, densely white villous, 6–13 cm long, 1–3 mm in diam.; blade green, asymmetric, widely ovate, 8.5–18 × 7–12 cm, rugose, usually with one obtuse lobe; base cordate, apex acuminate or acute, margin slightly denticulate, ciliate; adaxially green, densely tubercular-based setose, abaxially pale green, reddish when young, densely villous on veins; venation palmate, 5–6 primary veins, 2 or 3 secondary veins brunching dichotomous, tertiary veins reticulate. Inflorescence: dichasial cyme, peduncle 12–15 cm long, villous; bracts not seen. Staminate flower: pedicels 0.5–1 cm long; tepals 4; outer tepals 2, broadly ovate, 7–11 × 6–8 mm, apex rounded to acute, margin entire sometimes serrate and ciliate; inner tepals 2, oblanceolate, 5–7 × 1.8–2 mm, apex obtuse to acute, margin entire; androecium actinomorphic, stamens numerous; filaments longer than anthers, slightly fused at base; anthers yellow, 1–1.2 mm long, shorter than filaments, with two longitudinal slits. Pistillate flower: pedicel 1.2–2 cm long; tepals 2, white or pinkish, widely obovate to orbicular, 0.5–1 × 0.5–1 cm, apex acute, margin entire; styles 2–3, free; stigmas bilobed with twisted bands; ovary green or pinkish, trigonous ellipsoid, 1–1.5 cm long, 0.5–1 cm in diam. (wings excluded), white or red villous, 1-loculed; placentation parietal, placentae 3, 2-branched. Capsule pale green to pink, trigonous-ellipsoid, 1–1.5 cm long, 0.5–1 cm in diam. (wings excluded), villous, brownish when dried, 3-winged unequally; abaxial wing lunate, 0.9–1.2 × 0.3–0.7 mm; lateral wings lunate, 0.9–1.2× 0.2–0.5 cm. Seeds oblong, ca. 0.2 mm long.

#### Phenology.

Flowering in March–May, fruiting in April–June.

#### Etymology.

The epithet refers to the dense villous trichomes of the new species.

#### Habitat.

The species only grows on cliffs in limestone forests.

#### Distribution.

The species occurs exclusively in Ba Be National Park (Cho Ra county) of Bac Kan Province in Vietnam.

#### Note.

In Begonia sect. Coelocentrum, the new species is similar as *Begonia guangxiensis* in the dense hairy habit, mainly differing in the dense tubercular-based pubes on the abaxial surface of leaves (*vs.* villous trichomes in the latter species) and subequal wings (*vs.* unequal) (Wu and Ku 1997; Shui and Chen 2017). It is also similar as *B. calciphila* C.-I Peng in the shape and trichomes of leaves, but differs in 0.5–1.2 cm long stout inter-nodes (*vs.* 1.5–4.5 cm long slender inter-nodes) and hairy flowers and fruits (*vs.* glabrous in the latter species) (Peng et al. 2015b). In Bac Kan Province, Vietnam, there are four new species already described, viz. *B. regosula* Aver., *B. babeana* Aver. & H.Q. Nguyen (Averyanov and Nguyen 2012) and two more new species described in this paper.

**Figure 7.**
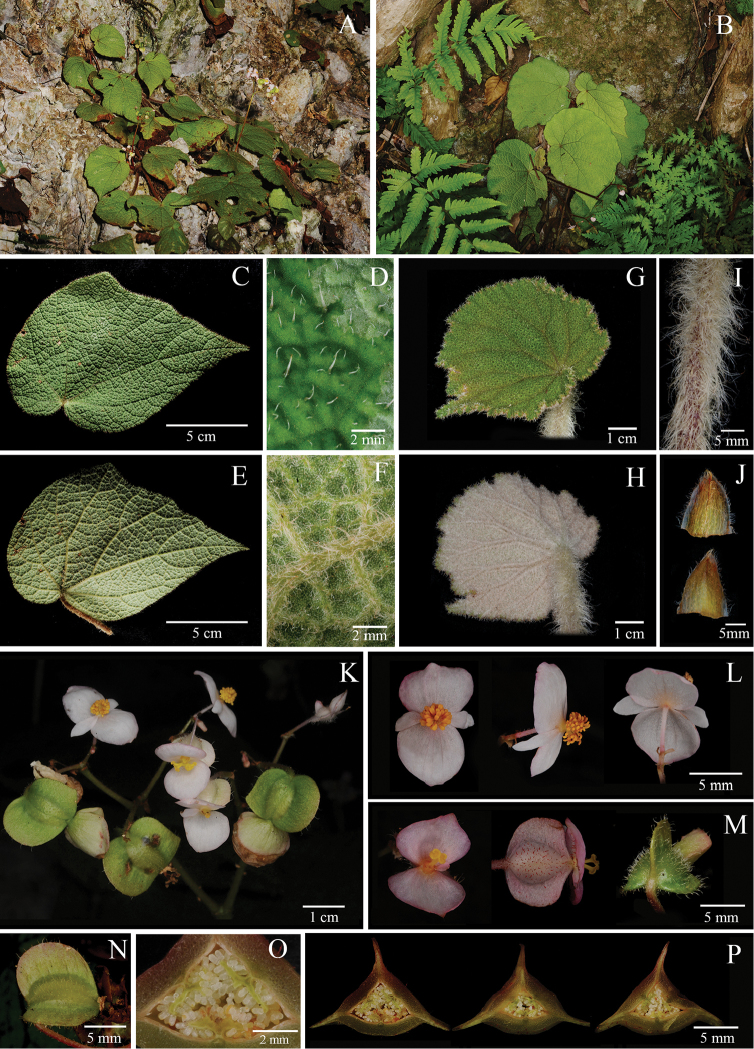
*Begonia mollissima* Y.M. Shui, H.Q. Nguyen & W.H. Chen **A** Habitat **B** Plant **C** View of adaxial leaf **D** Close-up of adaxial leaf **E** View of abaxial leaf **F** Close-up of abaxial leaf **G** Young leaf adaxially **H** Young leaf adaxially **I** Petiole **J** Stipule, adaxial view and abaxial view **K** Inflorescence **L** Staminate flower, face view, side view and dorsal view **M** Pistillate flower, face view, side view and dorsal view **N** Fruit, side view **O** Close-up of ovary cross section **P** Serial cross sections of ovary. (All photographs by Y.M. Shui).

### Begonia rhytidophylla

Taxon classificationPlantaeORDOFAMILIA

Y.M.Shui & W.H.Chen sp. nov.

urn:lsid:ipni.org:names:77175490-1

Fig. 8

Begonia sect. Coelocentrum Irmsch.

#### Remarks.

The new species is similar to *Begonia crystinilla* Y.M. Shui & W. H. Chen in the thick texture and shape of leaves, but differs in the flat adaxial surface of leaves with sparse setae (vs. the uneven with dense setae), pubescent abaxial surface of leaves (vs. lane), triangulate major wing of fruit (vs. semi-lunar).

#### Type.

CHINA. Guangxi Zhuang Autonomous Region, Jingxi county, Banliang community, 22°53'44"N, 106°26'22"E, 260 m a.s.l., at the roadside in bushes on the shady slope in the limestone hills, in fruits, 13 May 2017, *Y. M. Shui et al. B2017-300* (holotype, KUN).

#### Herb, rhizomatous.

Rhizome: stout, 7–10 cm long, 1–1.5 cm in diam. Stipule reddish, triangular, adaxially glabrous, abaxially villous, 1–1.5 × 0.5–0.7 cm. Leaves: petiole terete, dark red or brown, densely reddish expanding villous or strigose, 5–20 cm long, 0.5–0.7 cm in diam.; blade asymmetric, widely suborbicular to reniform, 7.5–14 × 6–11 cm; base cordate, apex subacute to obtuse; margin long ciliate; adaxially greenish, extremely sparsely short setulose; abaxially dark reddish, velutinous, densely strigose on reticulate veins; venation palmate, 5–6 primary veins, secondary veins brunching dichotomous, tertiary veins netted and obviously reticulate. Inflorescence: dichasial cyme, peduncle 13–16 cm long, villous; bracts caducous, broadly ovate to orbicular, margin serrate-ciliate, apex obtuse to rounded. Staminate flower: pedicel 0.6–1.0 cm long; tepals 3–4, white to pink, adaxially glabrous, abaxially hirsute-villous; outer tepals 2, broadly ovate, 1.2–1.5 × 1–1.3 cm, abaxially white to pale pink, glabrous to sparsely red setulose, base cuneate to rounded, apex obtuse to rounded, margin entire; inner tepals 2, white to pale pink, oblanceolate, 1–1.5 × 0.2–0.5 cm, base cuneate to rounded, apex acute to obtuse, margin entire; androecium actinomorphic, stamens numerous; filaments longer than anthers, slightly fused at base; anthers yellow, obovate, 0.5–1 mm long, apex truncate, shorter than filaments, with longitudinal slits. Pistillate flower: pedicel 1.5–2 cm long; tepals 3, pinkish or white; outer tepals 2, widely obovate to orbicular, 1.5–1.7 × 1.2–1.5 cm, base cuneate to rounded, apex rounded, margin entire; inner tepal 1, elliptic to oblanceolate, 1–1.3 × 0.2–0.4 cm, base cuneate to rounded, apex acute, margin entire; styles 3, fused at base; stigmas spirally twisted; ovary pinkish, trigonous ellipsoid, 0.6–1.2 cm long, 0.4–0.7 cm in diam. (wings excluded), red villous, 1-loculed; placentation parietal upper and axile at base. Capsule pinkish when fresh, brownish when dried, trigonous-ellipsoid, 1–1.5 mm long, 0.5–0.7 mm in diam. (wings excluded), 3-winged unequally; abaxial wing lunate, 0.6–1 ×0.3–0.6 cm; lateral wings narrowly lunate, 0.6–1 × 0.2–0.4 cm; Seeds oblong, ca. 0.2 mm long.

#### Phenology.

Flowering in August–November, fruiting in December–March next year.

#### Etymology.

The epithet refers to the reticulate pattern of nerves on the abaxial surface of leaves.

#### Habitat.

The species only grows on rocks at the entrance to caves or on the shady slope in limestone forests.

#### Distribution.

The species distributes to the border region between China and Vietnam, e.g. Jingxi county of Guangxi in China and Cao Bang and Tuyen Quang Province in Vietnam.

#### Additional examined specimens.

CHINA. Guangxi Zhuang Autonomous Region, Jingxi county, Renzhuang community, 13 May 2017, *Y. M. Shui et al. B2017-301* (KUN!). VIETNAM. Cao Bang Province, Tra Linh county, Quoc Toan committee, 22°45'56"N, 106°17'32"E, 11 April 2016, *Y.M. Shui, W.H. Chen, C. Liu, H.Q. Nguyen,H.T. Nguyen, N.Q. Chuong CK0953* (KUN!, CPC!); the same locality, Thang Moons mountain, 12 Apr 2016, *Y.M. Shui, W.H. Chen, C. Liu, H.Q. Nguyen,H.T. Nguyen, N.Q. Chuong CK1005* (KUN!, CPC!). Tuyen Quang province, Na Hang district, Sinh Long, 22°32'36"N, 105°23'42"E, 300 m a.s.l., 28 November 2017, flower pinkish, *Y.M. Shui, W.H. Chen, S.W. Guo, H.Q. Nguyen,H.T. Nguyen, N.Q. Chuong, K.S. Nguyen CK1453* (KUN! CPC!).

#### Note.

In Begonia sect. Coelocentrum Irmsch., the new species is unique in the dense reticulate nerves on the abaxial surface of leaves (Shui et al. 2002; Averyanov and Nguyen 2012). As to the morphology of leaves (thick texture, the even abaxial surface and broadly ovate lamina) and flowers (glabrous pink flowers and glandular hairs outside petals), the new species is similar to *B. crystallina*, *B. lanternaria* Irmsch. and *B. longgangensis* C.-I Peng & Yan Liu (Shui and Chen 2017). However, the latter three species are obviously different from the new species in their slightly pubescent abaxial surface of leaves. In Cao Bang Province, Vietnam, there already are the other three new species described recently (Peng et al. 2015a), viz. *B. caobangensis* C.-I Peng & C. W. Lin, *B. circularis* C.-I Peng & C. W. Lin and *B. melanobullata* C.-I Peng & C. W. Lin. The new proposed species is different from the above three species. During the survey in North Vietnam, *B. caobangensis* has been firstly discovered in the field in Tuyen Quang Province instead of Caobang Province. *B. circularis* seems to be similar as *B. lanternaria* Irmsch. but slightly different in the morphology of leaves and variable wings of fruit, while *B. melanobullata* is also similar to *B. nahangensis* Aver. & H.Q.Nguyen in Vietnam instead of *B. ferox* C.-I Peng & Yan Liu in China, but still slightly different with black hairs on the abaxial surface of leaves.

**Figure 8.**
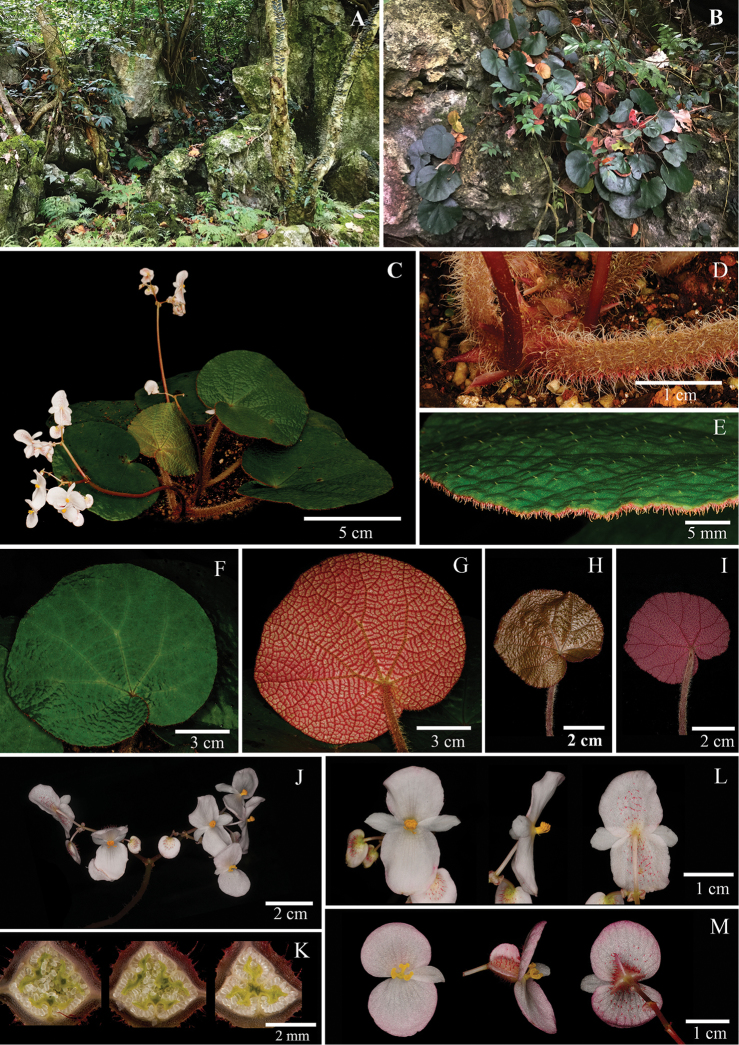
*Begonia rhytidophylla* Y.M. Shui & W.H. Chen **A** and **B** Habitat **C** Plant **D** Stipule and petiole **E** Close-up of adaxial leaf **F** View of adaxial leaf **G** View of abaxial leaf **H** Young leaf adaxially **I** Young leaf abaxially **J** Inflorescence **K** Serial cross sections of ovary **L** Staminate flower, face view, side view and dorsal view **M** Pistillate flower, face view, side view and dorsal view. (**A, B**, photographs by Y.M. Shui; **C–M** by S. Radbouchoom).

## Supplementary Material

XML Treatment for Begonia albopunctata

XML Treatment for Begonia bambusetorum

XML Treatment for Begonia erectocarpa

XML Treatment for Begonia gulongshanensis

XML Treatment for Begonia minissima

XML Treatment for Begonia mollissima

XML Treatment for Begonia rhytidophylla
